# Pattern of Paracetamol Poisoning: Influence on Outcome and Complications

**DOI:** 10.3390/toxics6040058

**Published:** 2018-09-29

**Authors:** Diego Castanares-Zapatero, Valérie Dinant, Ilaria Ruggiano, Harold Willem, Pierre-François Laterre, Philippe Hantson

**Affiliations:** 1 Department of Intensive Care, Cliniques St-Luc, Université catholique de Louvain, 1200 Brussels, Belgium; diego.castanares@uclouvain.be (D.C.-Z.); valerie.dinant@hotmail.be (V.D.); ilaria.ruggiano@student.uclouvain.be (I.R.); harold.willem@gmail.com (H.W.); pierre-francois.laterre@uclouvain.be (P.-F.L.); 2 Louvain Centre for Toxicology and Applied Pharmacology, 1200 Brussels, Belgium

**Keywords:** paracetamol poisoning, hepatic failure, outcome, critically ill patients

## Abstract

Acute paracetamol poisoning due to a single overdose may be effectively treated by the early administration of N-acetylcysteine (NAC) as an antidote. The prognosis may be different in the case of intoxication due to multiple ingestions or when the antidote is started with delay. The aim of this work was to investigate the outcome of paracetamol poisoning according to the pattern of ingestion and determine the factors associated with the outcome. We performed a retrospective analysis over the period 2007–2017 of the patients who were referred to a tertiary hospital for paracetamol-related hepatotoxicity. Inclusion criteria were: accidental or voluntary ingestion of paracetamol, delay for NAC therapy of 12 h or more, liver enzymes (ALT) >1000 IU/L on admission. Ninety patients were considered. Poisoned patients following multiple ingestion were significantly older (45 ± 12 vs. 33 ± 14) (*p* = 0.001), with a higher incidence of liver steatosis (*p* = 0.016) or chronic ethanol abuse (*p* = 0.04). In comparison with the subgroup of favorable outcome, the patients with poor outcome were older, had higher values for ALT, bilirubin, lactate, and lower values for factor V and arterial pH. In multivariate analysis, the arterial lactate value was associated with a bad prognosis (*p* < 0.02) (adjusted odds ratio 1.74 and CI 95:1.09–2.77). The risk of poor outcome was greater in the subgroup with staggered overdose (*p* = 0.02), which had a higher mortality rate (*p* = 0.01). This retrospective analysis illustrates the different population patterns of patients who were admitted for a single ingestion of a paracetamol overdose versus multiple ingestions. This last subgroup was mainly represented by older patients with additional risk factors for hepatotoxicity; arterial lactate was a good predictor of severity.

## 1. Introduction

The hepatotoxicity of excessive doses of paracetamol (acetaminophen) is a well-described entity, and risk factors are well identified. Paracetamol poisoning is now the leading cause of acute liver failure in the developed world [1]. Hepatotoxicity may be related to an excessive single dose in a previously healthy patient, but also to lower supratherapeutic or even therapeutic doses in patients who have a higher susceptibility to develop acute liver injury when exposed to paracetamol [2]. Most of the patients who are seen early after poisoning with a single paracetamol overdose can be safely managed in an emergency department. In contrast, the patients admitted after a significant delay or the patients who had ingested multiple supratherapeutic doses of paracetamol over several hours are at higher risk, since they have the possibility of developing acute liver injury despite the administration of the effective antidote, N-acetylcysteine (NAC) [3]. These patients will usually require intensive care management in a hospital with a liver transplantation program. The objective of this retrospective analysis was to review the medical charts of the patients who were admitted for paracetamol-induced hepatic injury in the intensive care unit of a university hospital with a large experience in liver transplantation. The first end point of the study was to investigate the influence of the pattern of paracetamol poisoning on outcome and complications by distinguishing between single ingestion and multiple ingestion. Single ingestion usually refers to suicidal ingestion, while multiple ingestion may either be related to suicide attempt (the so-called “staggered overdose”) or mainly to accidental ingestion (therapeutic error) of supratherapeutic doses for common pain relief. The second end point was to analyze the factors influencing the outcome.

## 2. Materials and Methods

We conducted over the period 2007–2017 a retrospective analysis of the patients referred to a tertiary, university teaching hospital (900 beds) with a diagnosis of paracetamol-induced hepatic injury. The transfer of the patients to our hospital was mainly justified by the hospital having a large experience in the field of liver transplantation. The inclusion criteria were as follows: all of the patients who had a documented history of an excessive paracetamol ingestion, either as a single dose as a suicide attempt, or in multiple doses over several hours, as the result of a suicide attempt (staggered overdose) or as the consequence of the use of supratherapeutic doses to control common painful disorders. The other condition for inclusion was a delay for the first administration of the effective antidote, NAC, that was equal to or greater than 12 h. This delay was calculated from the last ingestion of paracetamol, either in the case of single ingestion or after multiple ingestions. It was expressed in the following categories: 12 h, 12–24 h, 24–48 h, or >48 h. Among the biological criteria for inclusion, all of the patients had developed ALT >1000 IU/L at the time of ICU admission. Clinical symptoms on inclusion varied from normal physical examination to severe signs of hepatic failure or other organ dysfunction. The diagnosis of paracetamol-related hepatitis was made on the basis on a documented history and also on the determination of paracetamol blood concentration, with the limitation that paracetamol was no more detectable in some patients according to the delay since the last ingestion. The paracetamol blood concentration was plotted into the Rumack–Matthew nomogram to assess the risk of the development of severe hepatitis [4]. The estimated risk was based on the 150 µg/mL (4 h after ingestion) treatment line. The patients were also investigated by extensive laboratory toxicological investigations. Patients who had ingested another toxic substance that was able to induce liver injury were excluded from the analysis. Other causes of acute hepatitis (viral, neoplasic, ischemic…) were ruled out by the usual laboratory or radiological investigations. Patients with acute ethanol co-ingestion were excluded. For further analysis, the patients were divided in two subgroups: single overdose versus multiple ingestion. All of the patients were treated according to the same protocol for paracetamol poisoning. They received the 21-h NAC infusion regimen according to standard guidelines. For most of the patients who developed severe hepatic failure, the treatment was prolonged (100 mg/kg every 16 h) until paracetamol could be no more detected in the blood or until biological disorders (coagulation) had significantly improved together with the clinical recovery.

The following items were recorded and analyzed for the patients: demographics (age, gender), medical past history with a special attention to chronic ethanol abuse and pre-existing signs of liver steatosis (alcoholic or non-alcoholic), outcome expressed as death or survival, the presence of organ dysfunction (encephalopathy or coma, infections, acute kidney injury with the need for extrarenal epuration, the need for mechanical ventilation, for vasopressors).

### 2.1. Criteria for Liver Transplantation

The modified King’s College criteria were used for the detection of patients potentially requiring liver transplantation: (i) arterial lactate >3.5 mmol/L after early fluid resuscitation; (ii) arterial pH <7.3 or arterial blood lactate >3 mmol/L after adequate fluid resuscitation; or (iii) concurrent presence of serum creatinine >300 µmol/L, grade 3 or 4 encephalopathy and international normalized ratio (INR) >6.5 [5,6].

### 2.2. Biomarkers

Biological variables included the usual kidney and liver function tests, coagulation tests with INR and factor V, and arterial blood gas analysis, including lactate determination.

We calculated also the evolution of the AST/ALT ratio during the enzyme’s rise, and the velocity of AST and ALT decrease during recovery, which was expressed as the AST and ALT half-life calculated on serial blood determinations over time. The time to peak of AST/ALT was only calculated for patients with a single ingestion. Pooling all of the available measurements of AST and ALT concentrations, we estimated an area under the curve (AUC) during the enzyme’s release and recovery. Finally, we determined the paracetamol–aminotransferase product (APAP × AT) by multiplying the paracetamol concentration taken on presentation by the aminotransferase activity measured at the same time.

### 2.3. Statistical Analysis 

All of the analyses were conducted using SPSS 21 software (SPSS software [IBM Corp. 2011. IBM SPSS Statistics for Windows, Version 21.0. Armonk, NY, USA: IBM Corp]). Categorical variables were expressed as percentages, and continuous variables were expressed as mean ± one standard deviation (SD) or median with interquartile range (IQR). Categorical variables were analyzed using the Chi-squared test or Fisher’s exact test. Continuous variables were analyzed using an unpaired *t*-test or Mann–Whitney U-test, according to statistical distribution. The Kruskal–Wallis test was used to compare more than two groups. The data were subjected to the Kolmogorov–Smirnov normality test and Bartlett’s test for homogeneity of variance.

As the mortality rate was low in this series, we proposed as an alternative a comparison based on an index of severity (“bad outcome”) built from the combination of one or several of the following items: mortality, need for liver transplantation, mechanical ventilation, and length of ICU stay >10 days.

To establish risk factors of bad outcome, a univariate logistic regression analysis was performed to identify every variable associated with this issue. Validated variables were selected to be entered into a complete multivariate logistic regression model. Variable selection was performed with a method of forward elimination, using a *p*-value criterion of less than 0.20 for inclusion in the model. The results were expressed as an odds ratio (OR) with 95% confidence intervals (95CI). All of the tests were two-sided, with significance set at the 0.05 probability level.

Finally, receiver operating characteristic (ROC) curves were generated from significant variables in the univariate or multivariate analysis in order to assess their accuracy in predicting bad outcome.

### 2.4. Ethics and Consent

Institutional approval was provided by the Saint-Luc University Hospital Ethics Committee (Ref: 2018/07FEV/048), and our study complied with the Helsinki Declaration. To ensure confidentiality, patient data were anonymously recorded in the final database, in accordance with Belgian law. A waiver was obtained for written informed consent in view of the study’s retrospective design. 

## 3. Results

The main results are illustrated in Table 1, with a distinction between single and multiple ingestion. 

On the whole, 90 patients (53 women, 37 men) were included. For the patients admitted with a history of suicide attempt with a single dose, paracetamol blood concentration was above the 150-treatment line in 22 patients. Paracetamol was detectable in 14 patients (but interpretation was impossible due to a delay from ingestion greater than 24 h), and was theoretically in a non-toxic zone for four patients. The delay for NAC administration in the whole group was: 12 h (38%), 12–24 h (38%), 24–48 h (10%), >48 h (14%). In the whole group, the delay for NAC administration from the last paracetamol ingestion did not influence neither clinical nor biological variables (Table 2).

Seven fatalities occurred in the subgroup of multiple ingestion versus two in the single-ingestion subgroup. Five patients required liver transplantation in the subgroup with multiple ingestion versus two in the single-ingestion subgroup. Twenty-two patients fulfilled the modified King’s College criteria for liver transplantation, but only seven were effectively listed and transplanted. The modified King’s College criteria predicted death or a need for transplantation, with a positive predictive value of 100% and a negative predictive value of 87%. One patient was listed who did not receive a transplant due to the lack of graft availability, but fully recovered. The transplanted patients had a good outcome at one-year or five-year follow-ups when available. The analysis of the nine fatalities revealed that all of the patients were admitted with multiple organ failure. Only two patients had no additional risk factors for liver injury, while seven patients presented either a history of chronic ethanol abuse or a liver steatosis. In seven fatalities, the reason for the multiple ingestion of a supratherapeutic daily dose of paracetamol was the management of common pain. All of the patients fulfilled the King’s College criteria, but only three patients could be effectively listed for liver transplantation. For the other patients, the poor condition precluded the possibility of liver transplantation, and they were not listed. Two patients on the list died from neurological failure before a graft became available. One patient was effectively transplanted, but died on day 14 due to the absence of recovery of the initial multiple organ failure.

A comparison was performed between the subgroups with “bad outcome” and “good outcome” (Table 3).

Poor outcome was noted in 29% in the subgroup of patients with single ingestion versus 54% in the subgroup with multiple ingestions. The two subgroups differed for peak ALT, bilirubin, factor V, ammonia, minimal arterial pH, maximal lactate, AST/ALT ratio, t½ AST and ALAT, and APAP × AT. The patients with poor outcome were also significantly older (*p* = 0.005). Notably, among the patients presenting an accidental overdose following multiple ingestion, lactate values were higher in those who had a bad outcome (Lactate [Interquartile Ratio]: 5.2 mmol/L [1.4–3.4] versus 2.1 mmol/L [2.2–20], *p* = 0.001).

Finally, univariate and multivariate models were built to determine the factors associated with bad outcome (Table 4).

**Figure 1:**
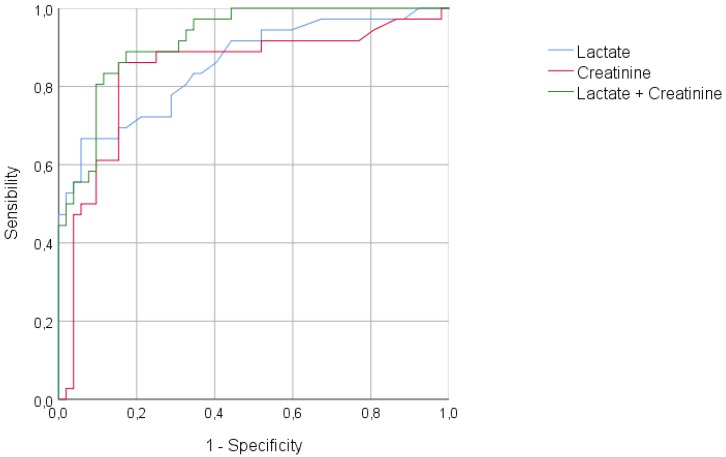
Receiver operating characteristic (ROC) curve for the prediction of bad outcome. Lactate, AUROC 0.86 (95CI: 0.77–0.94) (*p* < 0.001); creatinine, AUROC 0.84 (95CI: 0.74–0.93) (*p* < 0.001); lactate + creatinine, AUROC 0.92 (95CI: 0.87–0.97) (*p* < 0.001). AUROC: Area under the receiver operating characteristic (ROC) curve.

In the multivariate analysis, a statistically significant difference was observed for maximal lactate and creatinine. The analysis of the area under the curve (AUC) for receiver operating characteristics (ROC) curves confirmed the high predictive value of bad outcome provided by maximal lactate and creatinine values (Figure 1).

## 4. Discussion

This retrospective study aimed at analyzing the patients who experienced hepatotoxicity following paracetamol overdose after two different scenarios, either single or multiple ingestion. The common feature was a delay of 12 h or more for the initiation of NAC therapy. It is well documented that the majority of patients with an acute paracetamol overdose will not develop hepatotoxicity provided that NAC is administered within 8 h of overdose [7,8]. The occurrence of hepatotoxicity could be related to a delayed treatment, especially for patients with multiple ingestion, but it could also be related to factors that are linked directly to the patient’s pre-existing condition.

The use of the paracetamol treatment nomogram is restricted to patients with a known time of ingestion. It cannot be used after the following scenarios: staggered ingestions, delayed presentations (more than 24 h post-overdose), unknown time of ingestion, and repeated supratherapeutic ingestion. In most countries, the threshold for treatment is determined by a line starting at 150 mg/L at 4 h post-ingestion. From a large efficacy trial of oral NAC, a small risk (1%) of hepatotoxicity was determined for patients below this threshold [8]. However, despite early treatment with NAC, a small proportion of patients may still develop hepatotoxicity, with the reported incidence being up to 10% [9,10,11]. This suggests that some patients present additional risk factors to develop hepatotoxicity. Therefore, it was proposed to reduce the treatment line from 150 mg/L to a 100 mg/L for high-risk patients [12]. Nevertheless, hepatotoxicity could still occur in patients receiving NAC within 8 h of ingestion, despite a serum paracetamol concentration below the 100 mg/L nomogram treatment line [13]. This illustrates that paracetamol concentration alone is not a reliable risk predictor for hepatotoxicity.

Currently, there is no ideal risk prediction tool that identifies a patient’s risk of hepatotoxicity while being treated by NAC. In addition, there is no validated prediction tool to detect patients with a potential recovery or who will develop serious complications. Biomarkers were mainly used for single ingestion, with a time of presentation and initiation of NAC usually less than 8 h.

The paracetamol–aminotransferase multiplication product (APAP × AT) may be used in patients with later presentation, but with evident limitations. Indeed, a high multiplication product reflects early liver injury, which can be influenced by either a persistently high paracetamol concentration or a rising aminotransferase concentration [14]. The value of the paracetamol–aminotransferase product was also evaluated in patients with staggered, supratherapeutic, and delayed presentation overdoses, but interpretation should be cautious due to the small sample sizes of the populations [15,16]. A product with more than 1500 mg/L × IU/L after staggered, supratherapeutic, and delayed presentation overdoses was more predictive of hepatotoxicity than in patients with single acute overdose [3,17]. Our study confirms that patients with delayed NAC therapy and signs of hepatotoxicity had a paracetamol–aminotransferase product higher than 1500 mg/L × IU/L at admission.

The predictive value of changes in AST and ALT activities has also been questioned. An initial normal ALT or AST activity has a high negative predictive value of the subsequent development of hepatotoxicity in the patients who are treated with NAC [18]. An abnormal ALT or AST activity on presentation does not reliably predict the development of hepatotoxicity. According to the inclusion criteria, our patients had ALT activity higher than 1000 IU/L from admission. When looking at the AST and ALT kinetics, it has been shown that hepatotoxicity seemed more severe when the ratio AST:ALT exceeded 2:1 during the rising phase of the enzymes [19,20]. Two other items may also be taken into account: hepatic recovery seems to be indicated by an earlier fall in AST activity in comparison with ALT, and an AST/ALT ratio equal or less than 0.4 would also indicate recovery from hepatotoxicity [20].

The magnitude of liver enzymes release expressed by the AUC calculation from serial blood samples has not been investigated in other publications. The interpretation of our results is limited by a significant number of missing data either in the ascending or descending phase of enzymes release.

Blood lactate concentration is commonly raised in the patients with severe paracetamol-related acute liver failure. It is usually reflecting late presentation. In a series of 23 patients presenting more than 15 h after a paracetamol overdose, the median plasma lactate concentration was 3.6 mmol/L, and 11 patients (48%) had acidosis. Twenty patients (87%) developed hepatotoxicity, and four died of hepatic failure [21]. The increase in lactate concentration is mainly due to the reduction of hepatic clearance. We confirm the association between high lactate concentrations and poor prognosis. Lactate increase was also associated with a high incidence of shock and need for vasopressors [22]. Previous publications had also documented that a factor-V concentration of 10% or below was associated with a poor prognosis [23,24].

Our retrospective analysis is also outlining the importance of pre-existing risk factors, especially in patients who ingested supratherapeutic doses for pain relief. A retrospective study found an increased risk of hepatotoxicity after supratherapeutic doses of paracetamol in patients presenting non-alcoholic fatty liver disease (NAFLD) related to obesity [25]. The risk of acute liver injury is also increased after paracetamol overdose in patients with NAFLD [26]. By contrast, obesity alone does not seem to predispose to paracetamol-related hepatotoxicity [27]. The role of chronic ethanol consumption on paracetamol-related hepatotoxicity is still a matter of debate. Some retrospective studies did not find a significant difference in the severity of acute hepatitis among alcoholic and non-alcoholic patients when an overdose of paracetamol was taken [28,29,30]. A large retrospective study conducted on 533 patients who developed hepatotoxicity after either a single overdose or multiple ingestions failed to document a difference in outcome in chronic ethanol users [31]. However, this does not completely exclude the risk of hepatotoxicity of supratherapeutic doses of paracetamol in some predisposed chronic ethanol abusers.

Finally, our study did not investigate the possible influence of genetic polymorphism on acute liver failure following paracetamol overdose. Recently, it has been suggested that polymorphisms in the CD44 (rs1467558) and CYP3A5 (rs776746) genes might contribute to risk for paracetamol-induced acute liver failure, in addition to the previously discovered UGT1A-39UTR variant (rs8330) [32].

## 5. Conclusions

This case series illustrates that the pattern of paracetamol poisoning may have a significant influence on the outcome. Patients admitted after multiple ingestions developed more important cholestasis, renal failure, and arterial hyperlactatemia. Compared with the single-ingestion subgroup, staggered ingestion patients had a higher mortality rate and worse evolution, characterized by the requirement of mechanical ventilation or renal replacement therapy. Arterial lactate and serum creatinine were independent predictive factors of poor outcome, and their combination in a predictive model could be useful to identify patients at risk. Further prospective studies are warranted to confirm the impact of such a model on clinical management.

## Figures and Tables

**Table 1:** Main results in the subgroups with single or multiple ingestion. Legend: ICU, intensive care unit; CRRT, continuous renal replacement therapy; APAP × AT, paracetamol–aminotransferase product; AUC, area under the curve; NAC, N-acetylcysteine; AST, aspartate transaminase; ALT, alanine transaminase.

Variables	Measure	Single Ingestion (*n* = 55)	Multiple Ingestion (*n* = 35)	*p*-Value
Age (years)	mean, SD	33 ± 14	45 ± 12	0.001
Gender (M/F)	*n* (%)	20 M, 35 F	17 M, 18 F	0.25
*Outcome*				
ICU length of stay (days)	mean, SD	5.1 ± 1	6.9 ± 1.2	0.23
Mortality	*n* (%)	2 (3.6%)	7 (20%)	0.01
*Predisposing factors*				
Steatosis (Y/N)	*n* (%)	17 (31%)	20 (57%)	0.016
Ethanol abuse (Y/N)	*n* (%)	23 (42%)	26 (74%)	0.04
Obesity (Y/N)	*n* (%)	4 (7%)	2 (6%)	0.73
*Interventions–complications*				
Timing to NAC (h)	*n* (%)	12 h: 21 (38%)	11 (31%)	0.89
*(missing = 6)*		12–24 h: 19 (35%)	13 (37%)	
		24–48 h: 4 (7%)	4 (11%)	
		>48 h: 7 (13%)	5 (14%)	
CRRT (Y/N)	*n* (%)	12 (22%)	13 (37%)	0.12
Vasopressors (Y/N)	*n* (%)	8 (15%)	11 (31%)	0.06
Mechanical ventilation (Y/N)	*n* (%)	13 (24%)	14 (40%)	0.26
Infection (Y/N)	*n* (%)	11 (20%)	12 (34%)	0.14
*Indications for transplantation*				
Encephalopathy	*n* (%)	31 (56%) grade 0	14 (40%)	0.34
Grade (West-Haven)		14 (25%) grade 1	10 (28%)	
		1 (1.8%) grade 2	3 (9%)	
		3 (5.4%) grade 3	4(11%)	
		5 (9%) grade 4	4 (11%)	
Criteria for transplantation (Y/N)	*n* (%)	13 (24%)	9 (26%)	0.82
Transplantation (Y/N)	*n* (%)	5 (9%)	2 (6%)	0.7
*Biological markers*				
Peak AST (IU/L)	median (P25–P75)	9409 (5369–13,183)	10,176 (6644–15,285)	0.26
Peak ALT (IU/L)	median (P25–P75)	6250 (3110–9225)	7269 (5391–10,905)	0.14
Arterial pH	median (P25–P75)	7.38 (7.34–7.41)	7.35 (7.25–7.40)	0.006
Bilirubin max (mg/dL)	median (P25–P75)	9.7 (4.8–14)	12.4 (4.9–16)	0.047
Creatinine max (mg/dL)	median (P25–P75)	0.8 (0.6–1.5)	2.18 (0.8–4.4)	0.007
Lactate max (mmol/L)	median (P25–P75)	2.7 (1.8–6.5)	3.3 (1.6–8.3)	0.032
Factor min V (%)	median (P25–P75)	13 (4–26)	18 (9–48)	0.36
Ammonia (µg/dL)	median (P25–P75)	182 (126–302)	186 (138–354)	0.61
AST/ALT ratio >2	n (%)	4 (7)	9 (26)	0.017
t½ AST (h)	median (P25–P75)	39.6 (34.8–45.6)	38.4 (32.4–46.8)	0.58
t½ ALT (h)	median (P25–P75)	15.6 (14.4–20.4)	18 (14.4–25.2)	0.23
ny × AT (mg/L × IU/L)	median (P25–P75)	42,476 (23,746–93,680)	112,083 (34,926–270,910)	0.4
Time to peak ALT (h)	median (P25–P75)	60 (48–78)	42 (24–51)	0.006
AUC AST (IU·h·L^–1^)	median (P25–P75)	358,404 (230,076–527,568)	379,842 (230,521–726,090)	0.73
AUC ALT (IU·h·L^–1^)	median (P25–P75)	458,160 (287,336–583,344)	267,012 (115,720–470,274)	0.04

**Table 2:** Relationship between the delay of NAC therapy and biological variables and complications. Legend: APAP × AT, paracetamol–aminotransferase product; CRRT, continuous renal replacement therapy.

Variables (Median)	Time to NAC 12 h	Time to NAC 12–24 h	Time to NAC 24–48 h	Time to NAC >48 h	*p*-Value
	(*n* = 32)	(*n* = 32)	(*n* = 8)	(*n* = 12)	
Bilirubin max (mg/dL)	8.5	4.7	5	6.3	0.86
Peak AST (IU/L)	13,255	10,150	11,975	10,176	0.47
Peak ALT (IU/L)	7520	699	10,075	7050	0.16
APAP × AT (mg/L × IU/L)	48,538	58,658	29,520	70,403	0.96
Factor V min V (%)	15	12	13	41	0.1
Peak ammonia (µg/dL)	227	185	225	202	0.9
Arterial pH min	7.36	7.37	7.4	7.35	0.44
Lactate max (mmol/L)	3.5	3.3	2	2.9	0.78
Creatinine max (mg/dL)	1.04	1.43	1.13	1.85	0.36
Need for vasopressors	11 (34%)	8 (25%)	1 (12%)	0	0.1
CRRT	8 (25%)	10 (31%)	2 (25%)	4 (33%)	0.92
Mechanical ventilation	11 (34%)	11 (34%)	1 (12%)	5 (41%)	0.58
Infection	11 (34%)	5 (15%)	3 (37%)	4 (33%)	0.33
Mortality	6 (18%)	3 (9%)	0	0	0.2

**Table 3:** Analysis of variables in the subgroups with good or bad outcome. Legend: APAP × AT, paracetamol–aminotransferase product.

Variables	Good Outcome (*n* = 54)	Bad Outcome (*n* = 36)	*p*-Value
Age (year)	36 ± 11	42 ± 16	0.005
Peak AST (IU/L)	10430 ± 5227	12385 ± 7000	0.045
Peak ALT (IU/L)	8022 ± 3683	8109 ± 4782	0.81
Creatinine max (mg/dL)	2.2 (0.6–1.1)	3.2 (1.5–5.2)	<0.001
Bilirubin max (mg/dL)	3.6 (2.4–6.4)	7.5 (4.5–13.5)	<0.001
Factor V min (%)	17 (12–33)	10 (4–33)	0.021
Ammonia max (µg/dL)	161 (113–335)	264 (175–370)	0.001
Arterial pH min	7.4 (7.37–7.42)	7.32 (7.24–7.35)	<0.001
Lactate max (mmol/L)	2 (1.4–3)	6.8 (2.9–14.5)	<0.001
AST/ALT ratio >2	5 (9)	9 (25)	0.044
t½ AST (h)	42 (36–48)	34.8 (27.6–39.6)	0.005
t½ ALT (h)	15.2 (14–18.3)	18.3 (16–25.2)	0.001
APAP × AT (mg/L × IU/L)	31,414 (14,214–69,556)	79,990 (41,184–368,301)	0.001
Time to peak ALT (h)	60 (48–72)	60 (36–72)	0.11
AUC AST (IU·h·L^–1^)	354,324 (223,708–525,993)	388,512 (247,092–591,492)	0.11
AUC ALT (IU·h·L^–1^)	469,674 (290,662–539,460)	409,464 (191,958–654,102)	0.78

**Table 4:** Univariate and multivariate analysis to predict bad outcome. * Values were log-transformed. Legend: AUC, area under the curve.

Variables	Odds Ratio (95CI)	*p*-Value	Adjusted Odds Ratio (95CI)	*p*-Value
Lactate max	1.6 (1.26–2.2)	<0.001	1.74 (1.09–2.77)	0.02
Bilirubin max	1.61 (1.05–1.28)	0.002		
Creatinine max	1.71 (1.29–2.27)	<0.001	1.45 (1.05–1.97)	0.025
Single ingestion	0.36 (0.14–0.86)	0.022		
Age	1.05 (1.01–1.08)	0.007		
Gender (M)	1.17 (0.49–2.76)	0.72		
Factor V min	0.98 (0.96–1.01)	0.11		
Ammonia max	1.01 (1.002–1.01)	0.003	1.01 (0.99–1.02)	0.085
Alcohol	1.21 (0.51–2.82)	0.66		
Steatosis	2.10 (0.88–4.87)	0.1		
LOG peak AST *	3.76 (0.96–14.7)	0.06		
LOG peak ALT *	1.14 (0.99–3.34)	0.81		
AST/ALT >2	3.26 (0.99–10.7)	0.05		
t½ AST	0.56 (0.34–0.93)	0.026		
t½ ALT	3.89 (1.45–10.4)	0.007		
LOG APAP *	3.98 (1.77–8.92)	0.001		
Time to peak ALT	0.98 (0.96–1.004)	0.12		
LOG AUC AST *	1.6 (0.71–3.56)	0.25		
LOG AUC ALT *	1.04 (0.52–2.09)	0.91		
